# Fifteen-Fraction Radiosurgery Followed by Reduced-Dose Whole-Brain Irradiation With a Total Biologically Effective Dose of >90-100 Gy for a Locally Invasive Brain Metastasis From Lung Adenocarcinoma With a High Dissemination Potential

**DOI:** 10.7759/cureus.49596

**Published:** 2023-11-28

**Authors:** Kazuhiro Ohtakara, Fumiharu Ohka, Kuniaki Tanahashi, Takehiro Yamada, Kojiro Suzuki

**Affiliations:** 1 Department of Radiation Oncology, Kainan Hospital Aichi Prefectural Welfare Federation of Agricultural Cooperatives, Yatomi, JPN; 2 Department of Radiology, Aichi Medical University, Nagakute, JPN; 3 Department of Neurosurgery, Nagoya University Graduate School of Medicine, Nagoya, JPN; 4 Department of Neurosurgery, Gifu Prefectural Tajimi Hospital, Tajimi, JPN; 5 Department of Radiology, Nagoya University Hospital, Nagoya, JPN

**Keywords:** multileaf collimator, biologically effective dose, re-irradiation, oligo-metastases, large tumor, whole-brain radiotherapy, lung adenocarcinoma, stereotactic radiosurgery, fractionation, brain metastasis

## Abstract

A deep-seated, locally infiltrative 5.8-cm brain metastasis (BM) involving the ventricular wall and optic radiation is deemed unamenable for a safe total resection, while preventing tumor seeding. Meanwhile, radiotherapeutic management alone for such a BM close to the brainstem is also challenging. We describe such a BM (gross tumor volume [GTV] 40.3 cm^3^) from lung adenocarcinoma (LAC), located in the left temporo-occipital lobes, with extensive invasion to the tentorium cerebelli and a high potential for dissemination. The BM was treated with 15-fraction(s) (fr) stereotactic radiosurgery (SRS) followed by whole-brain irradiation (WBI) at 27 Gy/15 fr with a 19-day interval. During the SRS, the solid component away from the tentorium showed obvious shrinkage. The cumulative biologically effective doses (BEDs) of the minimum and D_99%_ of the GTV were ≥92.3 Gy and ≥102.6 Gy, respectively, where the BED was based on the linear-quadratic formula at an alpha/beta ratio of 10 (BED_10_). Despite a maximum response with nearly complete regression at 7.5 months, local tumor regrowth near the tentorial incisura became gradually apparent from 11.2 to 19.3 months. Salvage re-SRS with 53 Gy/10 fr specific to these lesions resulted in obvious regression at 5.8 months. However, radiation injury concomitant with triventriculomegaly progressed from 7.9 to 13.9 months, eventually leading to meningeal dissemination and patient mortality at 34.6 months. This case demonstrates that a BED_10_ ≥90-100 Gy in 30 fr to the GTV boundary with a more than two-week interval without combined systemic therapy is insufficient for achieving complete local tumor eradication of a 40-cc LAC-BM. Shorter treatment duration with a steeper dose gradient outside and inside the GTV in the SRS or a volumetric modulated arc-based SRS combined with simultaneously integrated WBI may improve efficacy and safety.

## Introduction

Brain metastases (BMs) without locoregional or any other distant tumor seeding are a pattern of recurrence after definitive surgery of non-small cell lung cancer (NSCLC) [1]. Local treatment such as external-beam radiotherapy (EBRT) or surgical resection is generally preferred over systemic therapy for oligo-BMs [2-4]. A single isolated BM requires long-term control (local curability) and safety with localized treatment, which can significantly affect prognosis [1,3].

Stereotactic radiosurgery (SRS) is preferred over whole-brain radiotherapy (WBRT) for relatively small and limited (oligo-) BMs [2,3]. In the meantime, surgical resection is prioritized over SRS for a BM with a >4-5 cm diameter, given the brain tolerance limits to SRS and occurrence of immediate decompression requirements [2]. A BM can reach >4-5 cm in diameter, with little to no relevant symptoms at diagnosis, depending on growth potential and location. These BMs, when infiltrating the brain surface and/or ventricular walls, have a high predisposition to develop meningeal/cerebrospinal fluid (CSF) dissemination, either naturally or iatrogenically [5]. A deep-seated, large BM that locally invades the ventricular wall and optic radiation is deemed unamenable for a safe total resection that preserves function and major vessels and precludes tumor seeding. Although a >5-fraction (fr) regimen has been adopted to perform SRS more effectively and safely for a large BM [6,7], proximity to the brainstem compromises sufficient target coverage with an adequate dose in SRS. Furthermore, a >25-30 cc BM is likely susceptible to adverse radiation effects (AREs), following ≤10-fr SRS [7]. WBRT that delivers ≥30 Gy, followed by a response-adaptive radiosurgical boost, can be considered as an alternative for such a large BM, especially including multiple BMs. However, older patients are more susceptible to acute and late detrimental effects, including neurocognitive decline [2,3].

Here, we have described a case of a minimally symptomatic, deep-seated 5.8-cm BM (40.3 cm^3^) infiltrating the ventricular wall, cortical surfaces, optic radiation, and tentorium cerebelli, with a high potential for dissemination. This BM developed 18.7 months after surgery for lung adenocarcinoma (LAC). The BM was treated with 15-fr SRS followed by a reduced-dose WBRT, with an interval of more than two weeks. The cumulative biologically effective dose (BED) was >90-100 Gy to the gross tumor margin, which resulted in nearly complete remission (CR) followed by partial tumor regrowth. The recurrent lesions required a repeat SRS 19.0 months after the prior SRS. We discuss ways to improve the efficacy and safety of radiotherapeutic management for the challenging BM.

## Case presentation

**Figure 1:**
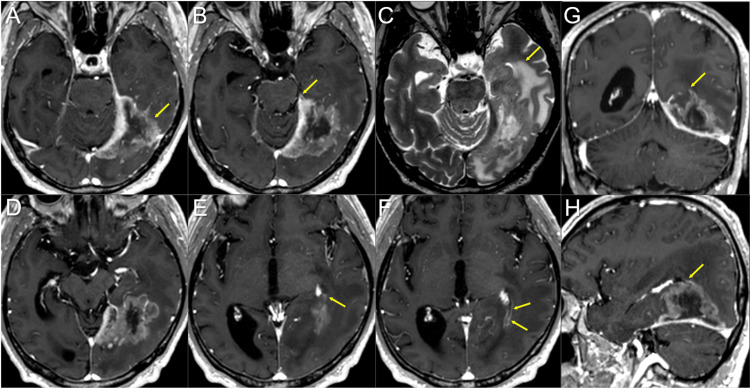
Magnetic resonance images before the upfront 15-fraction stereotactic radiosurgery for the brain metastasis The images show (A, B, D-H) contrast-enhanced T1-WIs, (C) a T2-WI, (A-F) axial images, (G) a coronal image and (H) a sagittal image. (A, B, D-H) These images are shown at the same magnification and coordinates under co-registration and fusions (B, C, the same cross-section; A, D-F, different cross-sections). A heterogeneously enhanced, indistinctly demarcated lesion (arrow in A) with a 58-mm maximum diameter is observed in the left temporo-occipital lobes, and functionally it involves the optic radiation. The mass attaches extensively to the markedly enhanced tentorium cerebelli that thickens toward the tentorial incisura. The extension of the lesion is proximal to the pons at the most ventral side of tentorial incisura (arrow in B). The lesion also infiltrates the trigone of the left lateral ventricle (arrows in E, G, H), and part of the ventricular wall is enhanced (arrows in F). (C) On the T2-WI, most of the lesion shows moderate high-intensity solid components with the unclear boundary with part of the brain parenchyma without edema. The lesion contains internal necrotic high-intensity components, along with the surrounding high-intensity parenchymal edema (arrow in C). WI: weighted image

This case involves a 66-year-old, right-handed, ex-smoker male who had been taking warfarin for 17 years for permanent atrial fibrillation and was noted to have an elevated carcinoembryonic antigen (CEA) level (11.3 ng/ml). The patient underwent right upper lobectomy for LAC (solid subtype, T1bN0M0) and left lower lobectomy for LAC (mixed subtype, T1bN0M0) 18.7 months and 5.7 years ago, respectively, in both of which major genetic alterations were unknown. Systemic examinations revealed a single, isolated, locally invasive mass lesion with a 5.8 cm maximum diameter in the left temporo-occipital lobes (Figure 1).

The only related symptoms were mild, occasional visual abnormalities on the right side (flashing) and intermittent left retro-orbital pain. The Karnofsky performance status (KPS) was 90%. Through multi-disciplinary deliberation, the intracranial lesion was initially treated with EBRT based on a clinico-radiological diagnosis of BM. Given the extensive invasion of the BM to the cortical surfaces, tentorium cerebelli, and ventricular wall, a high potential for dissemination was suspected (Figure 1). After fully considering the patient’s preference, we planned to perform 15-fr SRS followed by reduced-dose WBRT. The addition of WBRT using the dose equivalent to prophylactic cranial radiotherapy (25 Gy/10 fr) aimed at controlling any potential intracranial microscopic dissemination.

**Figure 2:**
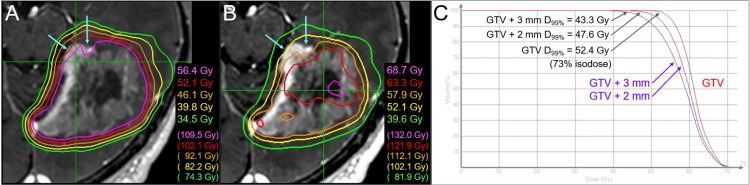
Dose distributions and DVHs for the upfront 15-fraction stereotactic radiosurgery The images show (A, B) representative isodose distributions superimposed onto axial contrast-enhanced T1-weighted image and (C) DVHs. (A, B) Nine representative isodose lines are shown to characterize the dose gradients mainly outside (A) and inside (B) the gross tumor boundary, along with the relevant BEDs based on the linear-quadratic formula with an alpha/beta ratio of 10 (BED_10_). The 52.1 Gy isodoses are shown in both, although the colors are different. The sufficient GTV coverages with 52.1-57.9 Gy are compromised near the brainstem (arrows in A, B). (C) The BED_10_s of 43.3 Gy, 47.6 Gy, and 52.4 Gy are 55.8 Gy, 62.7 Gy, and 70.7 Gy, respectively. The 52.4 Gy isodose is 73% relative to the maximum dose of the GTV. DVH: dose-volume histogram; BED: biologically effective dose; GTV: gross tumor volume; GTV + 2/3 mm: a structure generated by adding an isotropic 2/3 mm margin to a GTV boundary; D_99%_: a minimum dose encompassing at least 99% of the target volume

The gross tumor volume (GTV) for SRS was defined mainly on the enhancing lesion due to an unclear tumor-brain border on part of the BM in T2-weighted images (WIs) (Figure 1) [8]. The SRS was implemented using CyberKnife (CK)^®^ M6 treatment delivery system (Accuray Inc., Sunnyvale, CA), where a linear accelerator is mounted on an industrial robot with a six-axis manipulator arm, producing 6 MV flattening-filter-free (FFF) x-rays at a dose rate of 1000 MU/minute and featuring three secondary collimator types [6,9]. The basic planning scheme for the SRS was to encompass the GTV with ≥57.9 Gy (BED_10_ 80.2 Gy) as much as possible while ensuing ≤46 Gy (BED_2_ 116.5 Gy) of the maximum dose of the brainstem, where the BED_10/2_ was based on the linear-quadratic formula with an alpha/beta ratio of 10/2 [6,7]. The SRS plan was optimized using the InCise-2^TM^ multileaf collimator (MLC) (Accuray Inc.) with a 3.85-mm leaf width defined at a 800-mm source-to-axis distance and a maximum field size of 115 mm (leaf motion direction) × 100 mm, providing irregular shaped irradiation field [9]. The optimization algorithm was the CK-VOLO^®^ (Accuray Inc.), built into the dedicated planning system Precision^®^ (Accuray Inc.), with a finite-sized pencil-beam model for dose calculation (1-mm grid size) [6,9]. The estimated treatment time (EST) was 20 minutes per fraction, with 32 beams from 32 nodes with 41 segments. The dose distribution, dose-volume histograms (DVHs), and dosimetric parameters are shown in Figure 2 and Table 1.

**Table 1:** Planning parameters for the 15-fr radiosurgery *BED_10/2_ is a biologically effective dose based on the linear-quadratic formula with an alpha/beta ratio of 10/2; BED_10_ and BED_2_ are used as indicators of anti-tumor effects and late adverse effects to normal brain tissue, respectively. A total BED_10/2_ indicates the cumulative BED_10/2_ of the upfront 15-fr stereotactic radiosurgery and subsequent whole-brain radiotherapy in 27 Gy/15 fr. fr: fraction; GTV: gross tumor volume; GTV + 2/3 mm: GTV plus an isotropic 2/3 mm margin; D_max_: maximum dose; D_X%_: a minimum dose encompassing at least X% of the target volume; D_min_: minimum dose

Structures	Volumes	Parameters	Dose (BED_10_*)	Total BED_10_*	Total BED_2_*
GTV	40.30 cm^3^	D_max_	72.5 Gy (107.5 Gy)	139.4 Gy	299.0 Gy
		D_81.6%_	57.9 Gy (80.2 Gy)	112.1 Gy	220.9 Gy
		D_98%_	53.3 Gy (72.2 Gy)	104.1 Gy	199.3 Gy
		D_99%_	52.4 Gy (70.7 Gy)	102.6 Gy	195.2 Gy
		D_min_	46.2 Gy (60.4 Gy)	92.3 Gy	168.6 Gy
GTV + 2 mm	55.91 cm^3^	D_98%_	49.3 Gy (65.5 Gy)	97.4 Gy	181.6 Gy
		D_99%_	47.6 Gy (62.7 Gy)	94.6 Gy	174.4 Gy
		D_99.5%_	46.1 Gy (60.3 Gy)	92.1 Gy	168.2 Gy
GTV + 3 mm	66.94 cm^3^	D_98%_	45.3 Gy (59.0 Gy)	90.8 Gy	165.0 Gy
		D_99%_	43.3 Gy (55.8 Gy)	87.7 Gy	157.1 Gy
		D_99.7%_	39.6 Gy (50.1 Gy)	81.9 Gy	143.2 Gy

**Figure 3:**
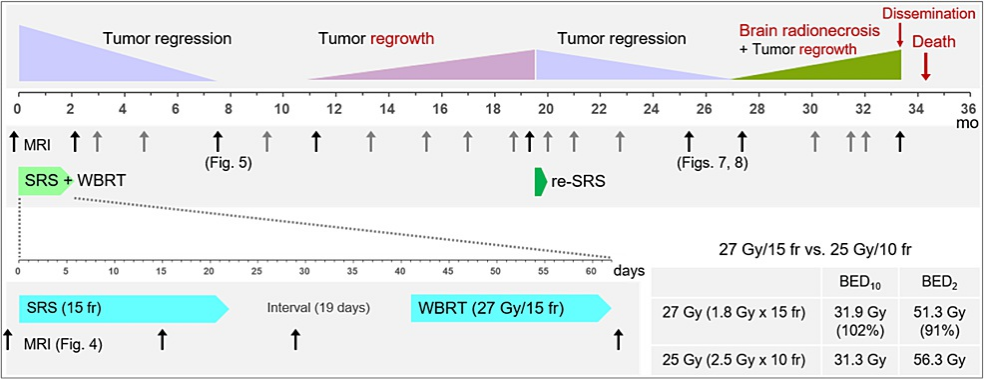
Summary of the radiotherapeutic management and subsequent clinical course The figure shows the initiation time of each external-beam radiotherapy, the durations, the timings of MRI evaluation, and the degrees and timings of treatment efficacies and relevant adverse effects. The arrows attached to the notation MRI indicate when the MR images were acquired, and the images from the times indicated by black arrows instead of gray are shown in later figures. The bottom right shows the differences in BED_10/2_s between 27 Gy/15 fr and 25 Gy/10 fr. Compared with 25 Gy/10 fr, 27 Gy/15 fr has a similar anti-tumor efficacy while 9% lower late effects. mo: months; MRI: magnetic resonance imaging; SRS: stereotactic radiosurgery; WBRT: whole-brain radiotherapy; fr: fraction(s); BED_10/2_: a biologically effective dose based on the linear-quadratic formula with an alpha/beta ratio of 10/2

The dose constraint to the brainstem substantially compromised the GTV coverage with 57.9 Gy, which was allowed considering the planned additional WBRT (Figure 3).

**Figure 4:**
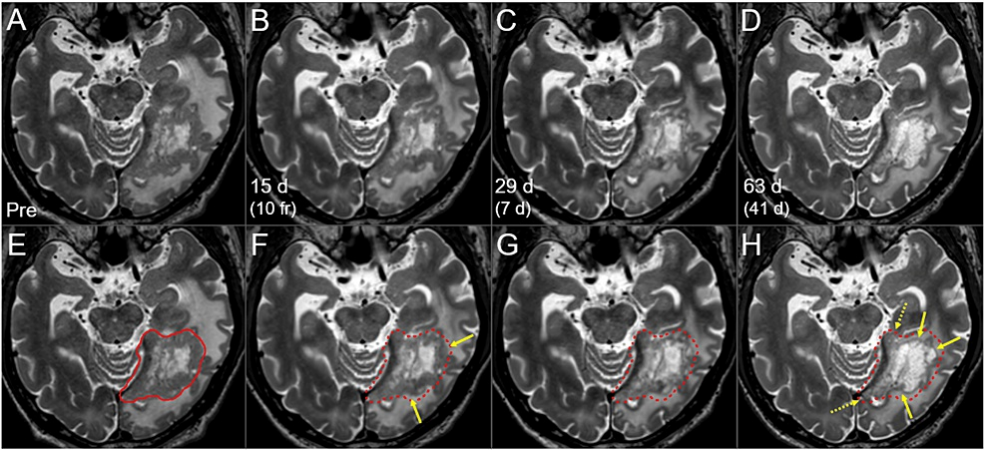
T2-WIs during the 15-fr radiosurgery and subsequent WBRT The images show (A-H) axial T2-WIs; (E-H) the GTV contour before the SRS (solid line in E, dashed lines in F-H) superimposed onto T2-WIs; (A, E) three days before the initiation of SRS; (B, F) 15 days after the SRS initiation (at 10 fr); (C, G) 29 days after the SRS initiation (seven days after the completion of SRS) and (D, H) 63 days after the SRS initiation (41 days after the SRS completion, the next day after the WBRT completion). (A-H) These images are shown at the same magnification and coordinates under co-registration and fusion based on the pre-SRS images. The solid component of the lesion gradually decreased (arrows in F, H), except for the components near the tentorial incisura (dashed arrows in H). d: days; WI: weighted image; GTV: gross tumor volume; SRS: stereotactic radiosurgery; fr: fraction(s); WBRT: whole-brain radiotherapy

T2-WIs were acquired appropriately for evaluating any change in the intracranial structures during the EBRT (Figures 3, 4) [6,10].

**Figure 5:**
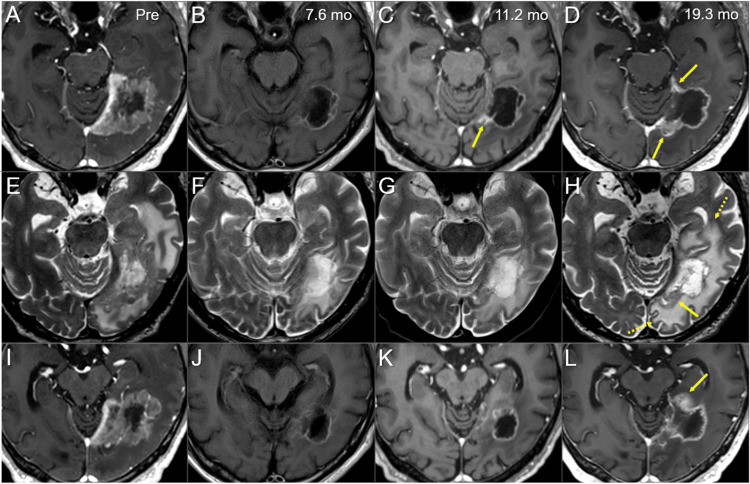
Magnetic resonance images before and after the radiosurgery followed by whole-brain radiotherapy The images show (A-D, I-L) axial CE T1-WIs, (E-H) axial T2-WIs; (A, E, I) before the SRS initiation, (B, F, J) at 7.6 mo after the SRS initiation, (C, G, K) at 11.2 mo and (D, H, L) at 19.3 mo. (A-L) These images are shown at the same magnification and coordinates under co-registration and fusion based on the pre-SRS images (A-H, the same cross-section; I-L, different cross-section). (B, F, J) The solid component, including the infiltrated region into the tentorium, almost completely regressed, leaving only slightly enhancing effects at the boundary. The perilesional edema decreased markedly, and the mass effects almost disappeared. (C, G, K) Part of the enhancing effects near the tentorial incisura slightly increased (arrow in C), which was deemed as adverse radiation effects at that time. (D, H, L) The enhancing lesions further enlarged (arrows in D, L), along with the corresponding mass on a T2-WI (arrow in H) and the increased perilesional edema (dashed arrows in H). CE: contrast-enhanced; WI: weighted image; SRS: stereotactic radiosurgery; mo: months

During the SRS, the solid component of the BM showed regression, especially in the regions unanchored to the tentorium. Tumor response during and one week after the SRS was consistent with that of LAC-BMs. Therefore, at more than two weeks (19 days) after the completion of SRS, WBRT in 27 Gy/15 fr was administered as planned. The dose per fraction was set to 1.8 Gy to attenuate both acute and late AREs relevant to WBRT while ensuring the anti-tumor efficacy similar to that of 25 Gy/10 fr (Figure 3). At the completion of WBRT, remarkable shrinkage and degeneration (higher intensity) of the solid component were observed. The EBRT was well tolerated, and oral betamethasone (1 mg) initiated six days before the SRS was tapered and discontinued 13 days after SRS completion due to a stable neurological condition. The lesion continued to shrink after the completion of EBRT, leading to nearly CR with only slight enhancement in the boundary at 7.6 months (Figure 5).

**Figure 6:**
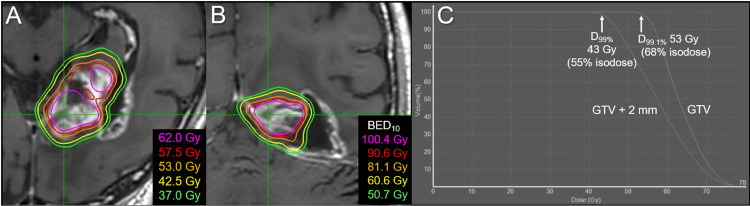
Dose distributions and DVHs for the 10-fraction re-radiosurgery limited to the regrowth lesions The images show (A, B) representative isodose distributions superimposed onto CE T1-WIs, (A) an axial image, (B) a coronal image and (C) DVHs. (A, B) Five physical isodose lines (A) are shown, along with the corresponding BED_10_s. (C) The BED_10_ of 43 Gy is 61.5 Gy. BED_10_: a biologically effective dose based on the linear-quadratic formula with an alpha/beta ratio of 10; D_X%_: a minimum dose encompassing at least X% of the target volume; GTV: gross tumor volume; CE: contrast-enhanced; WI: weighted image; DVH: dose-volume histogram

However, part of the enhancing rim near the tentorial incisura and the perilesional edema gradually increased from 11.2 to 19.3 months after the SRS (Figure 5), even while the neurological symptoms and the activity of daily living were stable. ^11^C-methionine positron emission tomography (PET) 17.2 months after the SRS showed increased uptake in the corresponding region, suggesting the existence of viable tumor tissue (images not shown) [11]. Therefore, the repeat SRS limited to the regrown lesions was performed (Figure 6, Table 2).

**Table 2:** Planning parameters for the 10-fraction re-radiosurgery BED_10/2_: a biologically effective dose based on the linear-quadratic formula with an alpha/beta ratio of 10/2; GTV: gross tumor volume; D_max_: maximum dose; D_X%_: a minimum dose encompassing at least X% of the target volume; GTV + 2 mm: GTV plus an isotropic 2-mm margin

Structures	Volumes	Parameters	Dose (BED_10_)	BED_2_
GTV	9.72 cm^3^	D_max_	78.4 Gy (139.9 Gy)	385.7 Gy
		D_85.6%_	57.6 Gy (90.8 Gy)	223.5 Gy
		D_98%_	54.7 Gy (84.6 Gy)	204.3 Gy
		D_99.1%_	53.0 Gy (81.1 Gy)	193.5 Gy
GTV + 2 mm	17.20 cm^3^	D_98%_	49.3 Gy (73.6 Gy)	170.8 Gy
		D_99%_	43.0 Gy (61.5 Gy)	135.5 Gy

**Figure 7:**
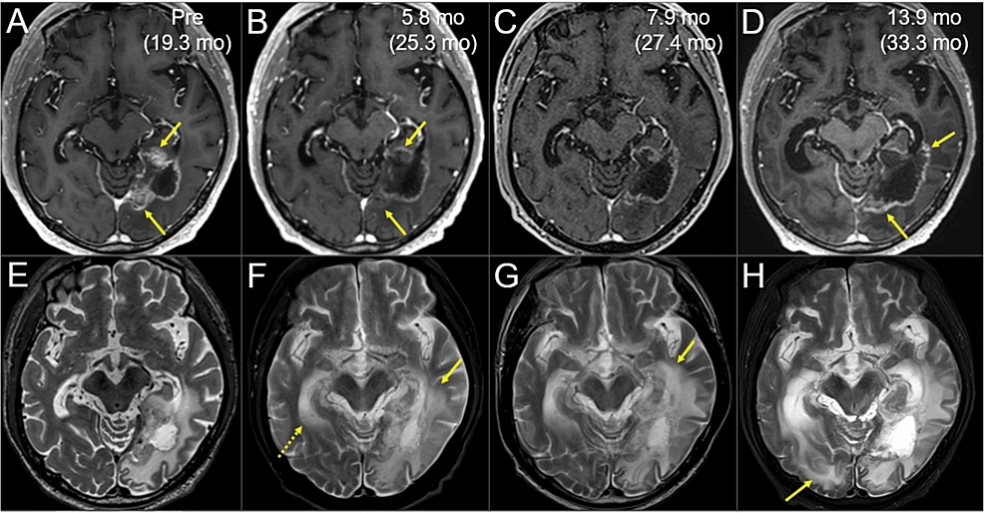
Magnetic resonance images before and after the re-radiosurgery, focusing on the local control of the regrowth lesions The images show (A-D) axial CE T1-WIs; (E-G) axial T2-WIs; (A, E) five days before the re-SRS (19.3 months [mo] after the initial SRS); (B, F) at 5.8 months after the initiation of re-SRS (25.3 months after the initial SRS); (C, G) at 7.9 months (27.4 months) and (D, H) at 13.9 months (33.3 months). (A-H) These images are shown at the same magnification and coordinates under co-registration and fusion based on the pre-SRS images. (A, B, E, F) The regrowth lesions (arrows in A) regressed substantially at 5.8 months (arrows in B), while the perilesional edema aggravated (arrow in F), and the temporal horn of the contralateral lateral ventricle enlarged (dashed arrow in F). (C, G) At 7.9 months, the regrowth lesions remained regressed, while the perilesional edema further increased (arrow in G). (D, H) At 13.9 months, the marginally enhancing lesion expanded (arrows in D), suggesting dominance of brain radionecrosis, along with aggravation of the perilesional edema (arrow in H). CE: contrast-enhanced; WI: weighted image; SRS: stereotactic radiosurgery; mo: months

**Figure 8:**
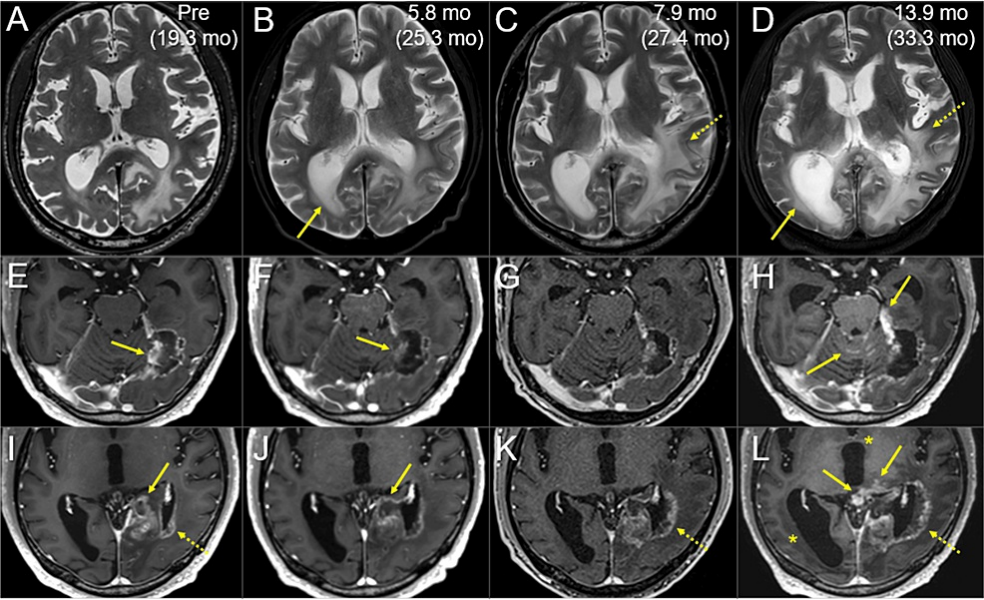
Magnetic resonance images before and after the re-radiosurgery, focusing on the progressions of radiation injury and tumor dissemination The images show (A-D) axial T2-WIs; (E-L) axial CE T1-WIs; (A, E, I) five days before the re-SRS (19.3 months after the initial SRS); (B, F, J) at 5.8 months after the re-SRS initiation (25.3 months after the initial SRS); (C, G, K) at 7.9 months (27.4 months) and (D, H, L) at 13.9 months (33.3 months). (A-L) These images are shown at the same magnification and coordinates under co-registration and fusion based on the pre-SRS images. The image series of A-D, E-H, and I-L each show different cross-sections. (A-D) The contralateral lateral ventricle dilated (arrow in B) at 5.8 months and progressed (arrow in D), along with aggravation of the perilesional/paraventricular edemas (dashed arrows in C, D). (E-L) Although the regrowth lesions almost regressed (arrows in E, F, I, J), meningeal dissemination appeared on the cerebellar surface and around the brainstem, along with the increased enhancement of the tentorial incisura (arrows in H, L). Brain radionecrosis around the left lateral ventricles progressed (dashed arrows in I, K, L), along with progression of triventriculomegaly (asterisks in L). WI: weighted image; CE: contrast-enhanced; SRS: stereotactic radiosurgery; mo: months

The GTV (9.72 cm^3^) for the re-SRS was defined based on T1/T2/PET matching. The planning design included sufficient GTV coverage with 53 Gy/10 fr (BED_10_ 81.1 Gy) and steep dose gradients [6,7,12]. The re-SRS was implemented with CK^®^ M6 using the Iris^TM^ Variable Aperture collimator (Accuray Inc.), which composes of two hexagonal banks of tungsten segments that produce dodecagonal apertures (virtually circular) ranging from 5 to 60 mm diameter [6]. The EST was 29 minutes per fraction, with 119 beams from 68 nodes. The regrowth lesions showed gradual regression after the re-SRS (Figures 7, 8).

However, from 5.8 to 13.9 months after the re-SRS, the development and progression of triventriculomegaly, aggravation of the perilesional and paraventricular edema, and an expansion of radionecrosis were observed [13]. Meningeal dissemination around the lesion, cerebellum, and brainstem was evident 13.9 months after the re-SRS. The patient developed unsteadiness and languid speech (28 months after the initial SRS), fatigue and dysgraphia (30 months), nausea, vomiting, difficulty in walking, and urinary incontinence (32 months), lumbago and urinary retention due to spinal dissemination (33 months) and a KPS decline of ≤60% (>33 months). The patient expired 34.2 months after the initial SRS (4.5 years after the second lung surgery) (Figure 3).

## Discussion

This case demonstrates that radiotherapeutic management alone with ingenuity can be an efficacious, safe, and minimally invasive treatment option for >5-cm (>40 cc) locally invasive BM from LAC when immediate decompression is unnecessary. With the aim of achieving long-term tumor control (if possible, complete tumor necrosis including microscopic invasion), the BED_10_ of >90-100 Gy was delivered in 30 fractions over two months with a 19-day interval. In addition, considering the dissemination potential, reduced-dose WBRT was sequentially combined as a precaution [14]. The biggest concern after treatment was whether AREs would be tolerable. However, the local residual tumors with subsequent regrowth were a major treatment failure, requiring the most reflection [11,15]. Subsequently, this required the re-SRS to the regions that had already been irradiated, reaching near the tolerable limit.

The viable tumor remnants mainly occurred on the medial side close to the tentorium. During the SRS, the solid component of the BM substantially decreased [6,10,11], while components anchored to the tentorium were immune to tumor shrinkage until the completion of EBRT (Figure 4). Significant tumor shrinkage during the SRS with an internal steep dose increase essentially led to a gradual dose increase in the GTV boundary [11,12]. However, the tumor components anchoring to the tentorium were less susceptible to tumor shrinkage and/or displacement beyond the original GTV boundary and to any tumor dose increase during the SRS.

The affected brain tolerated the re-SRS for approximately six months. However, radiation injury progressed thereafter, along with progressive ventriculomegaly that may have been attributed to CSF malabsorption caused by degeneration and/or necrosis of the tumor and parenchyma [13]. Re-SRS with a BED_10_ ≥80 Gy can be an efficacious and a safe option if the prior SRS dose was insufficient (BED_10_ <80 Gy) and the dose fractionation was appropriate considering volume effects [14-16]. However, re-SRS with partial targeting and insufficient coverage is no more effective than palliative therapy. Nevertheless, whether complete tumor eradication can be achieved with an initial treatment holds a key.

**Figure 9:**
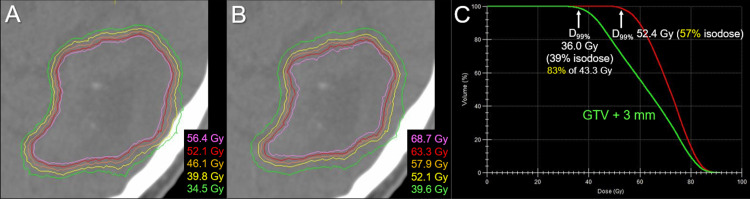
An alternative radiosurgical plan based on volumetric modulated arcs using a 5-mm leaf-width multileaf collimator The images show (A, B) the dose distributions superimposed onto an axial CT image and (C) the DVHs. (A, B) The representative isodoses are shown similarly to Figures 2A, 2B (the original plan). (A-C) The alternative plan for the initial SRS was generated using volumetric modulated arcs with the Agility^®^ collimator (Elekta AB, Stockholm, Sweden), where 52.4 Gy was assigned to the D_99%_ of the GTV as per the original plan. No constraint to the internal maximum dose of the GTV results in more concentrically laminated, steeper dose increase inside the GTV boundary (more inhomogeneous GTV dose) and more precipitous dose falloff (gradient) outside the GTV: the D_99%_ of the GTV + 3 mm (36.0 Gy) is reduced by 17% compared to that of the original plan (43.3 Gy). The cumulative BED_2_s of the GTV + 3 mm D_99%_ in the original and alternative plans are 157.1 Gy and 130.5 Gy (17% reduction), respectively. Thus, the alternative plan can attenuate the risk of relevant adverse radiation effects and provide room for GTV dose escalation without increasing the normal tissue dose outside the GTV. D_99%_: a minimum dose encompassing at least 99% of the target volume; GTV: gross tumor volume; GTV + 3 mm: GTV plus a uniform 3-mm margin; CT: computed tomography; DVH: dose-volume histogram; BED_10/2_: a biologically effective dose based on the linear-quadratic formula with an alpha/beta ratio of 10/2

The dose distribution of the initial SRS had a substantial scope for improvement in terms of dose conformity and steepness of the dose gradient outside and inside the GTV boundary (Figure 2) [12,17], although InCise2^TM^ MLC-based optimization significantly reduces irradiation time [9]. Optimization by combining relatively small and different sizes of the Iris^TM^ collimator likely generates a superior dose distribution for BMs [6,11,15]. In addition, volumetric modulated arcs (VMAs) using a general-purpose linac can provide an alternative plan with a more conformal and steeper dose gradient. This leads to a substantial dose increase inside the GTV boundary and dose reduction in the surrounding parenchyma (Figure 9) [12].

**Figure 10:**
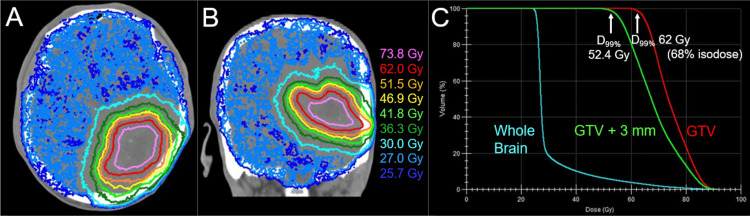
Another alternative plan with dose escalation to the gross tumor for 15-fr radiosurgery combined with simultaneously integrated reduced-dose whole-brain irradiation The images show (A, B) the dose distributions superimposed onto CT images, (A) an axial image, (B) a coronal image and (C) DVHs. (A-C) In the alternative plan, 62 Gy/15 fr is assigned to the D_99%_ of the GTV. The BED_10_ of 62 Gy/15 fr is 87.6 Gy, almost equivalent to a single dose of 25 Gy (BED_10_ 87.5 Gy). The cumulative BED_10_s of the GTV D_99%_ in the original and alternative plans are 102.6 Gy and 87.6 Gy (14.6% reduction), while the total treatment duration is reduced by approximately one-third with the alternative plan. The cumulative BED_2_s of the GTV D_99%_ in the original and alternative plans are 195.2 Gy and 190.1 Gy (2.6% reduction), respectively. The cumulative BED_2_s of the GTV + 3 mm D_99%_ in the original and alternative plans are 157.1 Gy and 143.9 Gy (8.4% reduction), respectively. D_99%_: a minimum dose encompassing at least 99% of the target volume; GTV: gross tumor volume; GTV + 3 mm: GTV plus a uniform 3-mm margin; CT: computed tomography; DVH: dose-volume histogram; fr: fraction(s); BED_10/2_: a biologically effective dose based on the linear-quadratic formula with an alpha/beta ratio of 10/2

Furthermore, VMAs enable simultaneously integrated use of reduced-dose WBRT, which can significantly reduce treatment time and escalate the GTV dose while reducing the surrounding brain dose (Figure 10) [14].

From 2021 onwards, we have primarily adopted the VMA-based SRS with or without simultaneous WBRT to irradiate more efficiently and ensure an earlier and more sufficient tumor response.

Sequential, systemic therapy based on examinations of major genetic alterations and programmed cell death ligand 1 (PD-L1) can be another treatment option to enhance the anti-tumor efficacy without dose escalation [4]. If the BM of this case harbored a driver gene alteration, the combined use of a tyrosine-kinase inhibitor might have augmented anti-tumor efficacy [18]. Even if targeted agents were not indicated, a bevacizumab-containing regimen could attenuate AREs relevant to local brain radionecrosis [19]. The significance of synergistic and/or sequential systemic therapy for isolated oligo-BMs remains to be investigated thoroughly.

The major limitations of this report include the unknown pathology of the intracranial lesion and unknown major genetic alterations from the initial diagnosis. These limitations hinder the ability to draw definitive conclusions. Given the unfavorable radiosensitivity, the lesion may have been pan-negative LAC with no major driver gene alterations. However, the necessity of conducting a pathological examination of the intracranial lesion via stereotactic biopsy remains controversial, given the inherent risk of iatrogenic tumor seeding. Nevertheless, this report sheds light on the establishment of efficacious and minimally invasive management for this challenging BM.

## Conclusions

Multi-fraction SRS followed by reduced-dose WBRT can be a minimally invasive and efficacious treatment option for a deep-seated, locally invasive, large LAC-BM with a high dissemination potential. Long-term local control (local tumor eradication) cannot be achieved in some LAC-BMs even when a BED_10_ ≥90-100 Gy is delivered to the GTV boundary over two months with a 19-day interval. Further dose escalation of the GTV and shorter treatment duration with a steeper dose gradient outside and inside the GTV in SRS or a VMA-based SRS with simultaneously integrated reduced-dose WBRT may improve efficacy and safety.
